# Medical students’ intrinsic versus extrinsic motivation to engage in research as preparation for residency

**DOI:** 10.1007/s40037-017-0388-3

**Published:** 2017-11-23

**Authors:** Belinda W. C. Ommering, Friedo W. Dekker

**Affiliations:** 1 Center for Innovation in Medical Education, Leiden University Medical Center, Leiden, The Netherlands; 2 Department of Clinical Epidemiology, Leiden University Medical Center, Leiden, The Netherlands

In this issue, Gupta and colleagues present interesting results on whether medical students who have written a scientific paper have a higher chance of being selected for a residency spot in paediatrics [1]. Before addressing the findings of their study and contemplating on extrinsic versus intrinsic motivation for conducting research, we would like to take a step back and elaborate on the overall importance of scientific training in medicine.

According to the CanMEDS, the core competencies of a scholar consist of two distinct elements: being able to *use* and being able to *conduct* research. This distinction between using and conducting research has also been adopted in the 2009 blueprint for medical education in the Netherlands [2].

*Using* research applies to all physicians, as every physician should be able to understand and use research to integrate scientific knowledge in clinical practice and form grounded diagnosis [3, 4]. Involving scientific knowledge in clinical decisions requires physicians to keep up-to-date with the newest developments in medicine. Also, they should be able to critically appraise scientific literature and discuss research findings both with colleagues, and with patients who have better access to information from the internet than in the past [5, 6]. Moreover, using research can also be seen as a contribution to the process of life-long learning, continuously translating new knowledge into patient care [7].

In addition to all physicians utilizing research, medical education also intends to cultivate some physicians who will *conduct* research. These physician-scientists are needed to make progress in the continuously evolving field of medicine and to form a bridge between science and practice, by translating research outcomes into clinical settings. Furthermore, physician-scientists have the ability to identify relevant clinical questions and problems. By being actively engaged in clinical practice, these physician-scientists encounter daily clinical questions and problems, which can serve as inspiration for scientific research. Moreover, physician-scientists can contribute to developing new knowledge by formulating research questions, thinking about proper study designs, contributing to data collection and interpretation of results, and writing scientific papers. They can also develop clinical guidelines, and are role models by implementing this new knowledge into clinical practice [8, 9]. In this process of translational research, back and forth from ‘bench to bedside’, physician-scientists are key. However, in the last decade concerns have been raised about the declining number of physician-scientists in many countries [3, 10, 11].

In order to train all physicians to reach the required level of a scholar, and also to counteract the decline of the physician-scientist workforce, scientific training must play an important role in medical education. This can be established in two ways. The first is by engaging students with talents and ambition into extracurricular research programs. Examples of these extracurricular research programs are MD/PhD programs and scholarly concentration programs, the latter becoming a common method within the field of medical education [12, 13]. The second is by integrating research into medical curricula in a way that it reaches all medical students [7]. We believe that acquainting students with both using and conducting research should already be clear goals in the early phases of medical education. Actually conducting research can help students to understand and use research, while at the same time this offers an opportunity to shed light on a possible research-oriented career. Also, it helps to recognize talented students and helps them develop into the next generation of physician-scientists.

Healey and his colleagues have developed a framework to illustrate the research-teaching nexus and explain four ways in which students in higher education can experience research in the curriculum. According to this framework, students can be viewed as audience or as active participants, while the emphasis can be on the research process or on research content. It has been argued that viewing students as participants, combined with an emphasis on the research content, is a form of active learning [14]. Active learning, or ‘learning by doing’, is seen as the most optimal way to engage students in this kind of activity [15–17]. This can be established, for instance, by offering students the opportunity to conduct or participate in an authentic research project during their medical training. In this respect, the hands-on experience of publishing a scientific paper may well be seen as an excellent example of active learning, and thus would be a powerful means to cultivate scientific minds.

In this issue, Gupta and colleagues show thought-provoking research findings, which indicate that pre-residency publication is not associated with achieving a higher rank in first-choice match for paediatric residency in Canada [1]. From this, they conclude that extrinsic motivation should not be the main driving factor for doing research and publishing a paper, as apparently a published paper does not help to get a higher ranking. They also argue that educators should be honest about this to students and adapt the information communicated towards them. Of course, the question can be raised whether these findings tell us something about the intrinsic value of writing a publication in medical training? Perhaps they tell us more about the selection procedure in this specific paediatric setting in Canada, and the weight given to publications by selection committees, which can vary between specialties and even countries. Imagine what could happen if medical students were to read Gupta’s paper and indeed no longer have the ambition to do research and publish a paper? Or, the other way around, what if students who are interested in doing research no longer apply for a residency in paediatrics? What might be the consequences for the scientific image of paediatrics and other specialties alike?

Nevertheless, as educators we are happy to see that extrinsic motivation for doing research should be less important for medical students, making room for developing sincere intrinsic motivation. In line with this, we would like to cordially invite educators to emphasize the importance of research for the future of clinical practice and patients, thereby fostering the intrinsic motivation of these students. Studies based on the Self-Determination Theory have shown that intrinsic motivation is related to better academic performance and general wellbeing [18, 19]. Hence, educators should aim to stimulate students’ intrinsic motivation. However, we can imagine that students tend to start participating in research because of extrinsic motivators, such as the belief that conducting research will secure a competitive residency spot or broaden their future career options [3, 20–22]. This notion of the importance of research seems logical, as many educators emphasize the need to do something extra to students from the beginning of medical training.

But is conducting research because of these extrinsic incentives always bad? Could it be that students are extrinsically motivated when they start conducting research, but become intrinsically motivated along the way? Perhaps students become more and more familiar with the ins and outs of research, and discover how much fun it is to do. By engaging in research, students could also discover their own talents and research competencies, which contributes to their intrinsic motivation for research. For instance, experiencing research could enhance students’ research self-efficacy over time, and according to Bandura’s Social Cognitive Theory enhanced self-efficacy influences intrinsic motivation [23]. This is in line with one component of the Self-Determination Theory, which states that perceived competence of a person is related to his or her intrinsic motivation. Moreover, students can shift in the continuum of the different types of motivation, as also described by the Self-Determination Theory [18, 19]. Thus, it seems fair to assume that a process of transitioning extrinsic motivation into intrinsic motivation over time is possible. Before students are discouraged from doing research because it probably has no value in gaining a residency spot or a first match outcome, we believe it is of great importance to investigate whether this extrinsic motivation to do research can indeed turn into feelings of intrinsic motivation while being actively engaged in research.

In conclusion, we would like to emphasize the great importance of directing a sufficient number of medical students towards a research-oriented career. This could help to avoid a further decline in physician-scientists and thereby contribute to the next generation of physician-scientists that is so urgently needed in our world of medicine. It would be unfortunate for every specialty, including paediatrics, if students were to engage less and less in research before entering residency. Therefore, dealing with the intrinsic *and* extrinsic feelings of students towards research in such a way that could trigger their intrinsic motivation for research could be of great value.
